# Theoretical Basis for Switching a Kramers Single Molecular Magnet by Circularly-Polarized Radiation

**DOI:** 10.3390/ma12233865

**Published:** 2019-11-22

**Authors:** Alexander G. Maryasov, Michael K. Bowman, Matvey V. Fedin, Sergey L. Veber

**Affiliations:** 1 Voevodsky Institute of Chemical Kinetics and Combustion of the Siberian Branch of the Russian Academy of Sciences, 630090 Novosibirsk, Russia; 2 Department of Chemistry & Biochemistry, The University of Alabama, Tuscaloosa, AL 35487, USA; mkbowman@ua.edu; 3 Novosibirsk Institute of Organic Chemistry of the Siberian Branch of the Russian Academy of Sciences, 630090 Novosibirsk, Russia; 4 International Tomography Center of the Siberian Branch of the Russian Academy of Sciences, 630090 Novosibirsk, Russia; mfedin@tomo.nsc.ru; 5 Department of Physics, Novosibirsk State University, 630090 Novosibirsk, Russia

**Keywords:** Kramers ion, circular polarization, selective transition, zero field splitting, far infrared spectroscopy, SMM, magnetization inversion

## Abstract

The *d*-group Kramers ions, having strong zero field splitting (ZFS) with axial symmetry and a negative D value for the ZFS Hamiltonian, are widely considered as candidates for use as single molecular magnets (SMMs). An important need is the means to switch the SMM between its states in a reasonably short and predictable period of time, which is generally not available. We propose an approach, Zeeman–far infrared (ZeFIR) double resonance, in which circularly polarized alternating magnetic fields in the far infrared (FIR) range induce selective magnetic dipole transitions between different Kramers doublets of the SMM and polarized microwave (mw) pulses transfer excitation inside the upper Kramers doublet. A combination of FIR and mw pulses allows unidirectional switching between +*S* and −*S* states of the ion. The proposed approach is considered for a model quartet system with total spin *S* = 3/2, which seems to be the most promising object for selective resonance manipulations of its states by circularly polarized radiation.

## 1. Introduction

Single molecular magnets (SMM) [1,2] are molecular objects with a high-spin (S>1) ground state, strong zero field splitting (ZFS) interaction with D<0, and slow magnetic relaxation. The lowest-energy states of an SMM are a pair of highly-magnetized levels with magnetic quantum numbers m=+S and −S and long lifetimes. For many uses of SMMs, for example, as molecular magnetic memory or as gates, there must be an efficient way to switch them between these two states that is rapid and precise, and can be performed in a known period of time. In the area of molecular magnetism, several ways have been proposed for switching magnetic properties at the molecular level. Most of these methods consider switching between high-spin and low-spin electronic states, so-called spin crossover (SCO) [3,4], rather than between specific quantum levels of an SMM. Several mechanical and chemical means to switch between spin crossover states have been reviewed [5], but in these cases, the speed of their switching is too low for most applications. Pulsed magnetic fields could provide ultrafast switching, but exceed present technologies, for example, requiring sub-picosecond pulsed magnetic fields of >35 Tesla [6]. Light-induced spin state switching [7,8,9,10] has been demonstrated, but the need to dissipate the energy absorbed from optical photons would limit the throughput of such devices [11,12].

Techniques utilizing SCO transitions have limited utility for SMM state switching. They change the SMM spin state S, but without selectivity for the magnetic quantum number m. Thus, they could erase or initialize memory, but not, for example, write a value to memory. Quantum tunneling can switch between quantum levels of an SMM [13,14], but is stochastic and difficult to control. Even a recent elegant demonstration of reading the SMM states of holmium atoms on a magnesium oxide surface was forced to write those states using repeated attempts until the desired SMM value stochastically appeared [15]. If writing a value to memory remains a stochastic process, the write operation will take an indeterminate amount of time, making it difficult to schedule operations involving SMM logic and memory. The processes of photon-assisted tunneling could be introduced into the algorithm of SMM switching, as was shown on the example of *S* = 10 Fe_8_ single-molecule magnet [16]. The use of circularly-polarized light was proposed for selectively inducing a transition either between the *S* and (*S* − 1) states or between the −*S* and (1 − *S*) states, expecting further tunneling to the −*S* state or +S state, respectively. This approach is limited by the finite rate of the tunneling process and by the single quantum relaxation transition to the initial state. Such circularly polarized ac magnetic fields have been suggested for use in quantum computing algorithms for the effective creation of entangled states [17].

In this contribution, we considered in detail the use of circularly polarized far infrared (FIR) and microwave (mw) radiation pulses for selective switching of a *S* = 3/2 SMM between its +S and −S ground states. The influence of the spin system imperfectness to the switching efficiency was discussed. The theoretical calculations performed predict the possibility of unidirectional switching within tens of nanoseconds using existing sources of FIR and mw radiation.

## 2. The Approach

We propose a combination of FIR and mw pulses [18,19,20] using circularly-polarized radiation for reliable, selective, unidirectional switching between the states of a high-spin, Kramers *d*-ion SMM. We discuss this novel multifrequency method in the context of a model 3d^7^ Co(II)-based SMM with a large, nearly axially-symmetric [21] ZFS interaction with |E|≪|D|. Its ground state for D<0 would be a spin S=3/2 quartet with two Kramers doublets and only the doublet having large magnetic dipoles would be populated at low temperatures. The unidirectional switching of the SMM proposed here is widely applicable across the family of SMMs with S≥3/2. However, S=3/2 SMMs provide a simple (with the lowest number of transition frequencies possible), yet realistic model for illustrating the principles underlying unidirectional switching. A useable SMM or information storage unit must remain in one state for a long time, yet must be switched to the other system state quickly and reliably when needed. These are opposing requirements, because facile switching generally accompanies fast relaxation and short lifetimes caused by thermal or other random fluctuations. In this aspect, the two states of the Kramers doublet are good candidates for information storage because they achieve long lifetimes owing to Kramers theorem and time-reversal symmetry, which make it impossible for an electric field to cause a transition or relaxation within the Kramers doublet [22]. It should be mentioned here that non-Kramers systems having an integer spin value are affected by electrical fields, which can even remove level degeneracy when E≠0 and can mix +S and −S sublevels directly, thus making non-Kramers systems poorer SMM candidates. However, as mentioned above, the fundamental reason for the long lifetimes of the two states of a Kramers doublet leaves few possibilities for manipulating or switching the system state. One way is to use a nonresonant external magnetic field $B_{0}$, but that is generally not very rapid or spatially addressable. Another is to manipulate relaxation between the states thermally, but this leads to an equilibrium mixture of states, and not to switching to a specific state. The approach proposed here uses circularly-polarized magnetic dipole transitions involving interdoublet transitions (in the following, referred to as FIR transitions emphasizing typical values of ZFS in considered systems, see Section 3.1) with additional control by conventional electron paramagnetic resonance (EPR) transitions to switch SMMs rapidly and selectively between states. The practical realization of this approach requires the use of a pulsed EPR spectrometer combined with an option to irradiate pulsed irradiation of the sample by FIR light, similar to the continuous-wave EPR setup reported earlier [23]. Let us note that the proposed technique is actually a type of double resonance method, which can be called Zeeman–far infrared (ZeFIR) double resonance. Below, we describe the structure of the system energy levels using the spin Hamiltonian approach and analyze the system evolution under the action of radiation pulses using the density matrix technique [24].

## 3. Results and Discussion

### 3.1. The Model System and its Hamiltonian

The model system is an axially-symmetric S=3/2 quartet with nuclear spins neglected. An external magnetic field is applied along the molecular symmetry axis, Z, which coincides with the unique principal axis of the ZFS and g-tensor. The FIR and mw radiation propagate along Z with their magnetic fields oscillating perpendicular to Z. The static spin Hamiltonian of such a system is

(1) $${\hat{H}}_{0}=D\left({\hat{S}}_{Z}^{2}-S\left(S+1\right)/3\right)+E\left({\hat{S}}_{X}^{2}-{\hat{S}}_{Y}^{2}\right)+\omega_{0}{\hat{S}}_{Z},$$

where $\omega_{0}=g_{∥}\beta B_{0}/\hbar$, with $B_{0}$ as the external magnetic field strength, β as the Bohr magneton, ħ as Planck’s constant, and $g_{∥}$ as the principal value of the ‘true’ g-tensor [25] of the quartet along  Z. For convenience, the principal values of g are treated as positive. The eigenfunctions, $\psi_{m}=|m\rangle$, of ${\hat{H}}_{0}$ are also those of ${\hat{S}}_{Z}$ because, in the case of axial symmetry, E≈0, and these are numbered by the spin projection, m=±3/2,±1/2, onto Z. Let us note that, for axial symmetry of spin Hamiltonian (1), the symmetry of ligands surrounding, for example, the Co^+2^ ion, should be C_3v_ or higher [22]. The relative energy of the spin states (eigenvalues, in units of ħ) from the spin dependent terms of the Hamiltonian (1), are

(2) $$E_{m}/\hbar =\omega_{m}=D\left(m^{2}-5/4\right)+\omega_{0}m.$$

**Figure 1:**
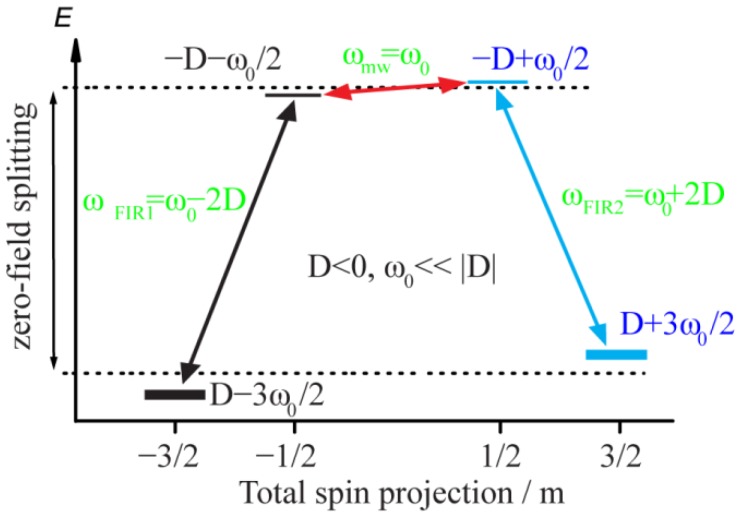
Energy levels of the model system in an applied magnetic field. Black and blue horizontal lines represent levels with negative and positive m, respectively. The arrows denote allowed transitions, the red arrow is the electron paramagnetic resonance (EPR) transition of the excited Kramers doublet, and the green labels indicate transition frequencies.

This system of four levels has six possible transitions: three single quantum (Δm=±1) transitions that are allowed for excitation by an oscillating magnetic field perpendicular to Z; but two double quantum (Δm=±2) transitions and one triple quantum (Δm=±3) transition that are forbidden for excitation by electric or magnetic fields. The allowed transition within the m=±1/2 Kramers doublet is the EPR transition at $\omega_{0}$, however, at low temperatures, when kT≪2|D|ℏ, the equilibrium populations may be so small that the EPR signal is undetectable [26]. The forbidden triple quantum transition at $3\omega_{0}$ within the m=±3/2 Kramers doublet has an effective $g≅3g_{∥}$ [27] and only becomes very slightly allowed when E≠0 [27]. The remaining two allowed transitions are between states having the same sign of m, with transition frequencies $\omega_{0}\pm 2D$ as large as several Terahertz [28,29,30]; Figure 1. These two interdoublet or FIR transitions are well-resolved in energy from the transitions within each Kramers doublet.

At zero magnetic field, the transition within the m=±3/2 Kramers doublet is strictly forbidden, making them (in theory) extremely long-lived. A 2|D| barrier blocks thermally activated relaxation between those levels. Thus, the S=3/2 system with large axial ZFS and negative D could be considered as an SMM prototype.

It is important to note that we take the positive direction of the Z axis as the same as for the external magnetic field (to avoid mixing the m=±1/2 states within the excited Kramers doublet by the magnetic field). In that case, the energy within each Kramers doublet increases with the quantum number m, as it does for the spin of a free electron. This means that the EPR transition between the m=±1/2 levels is induced by circularly-polarized mw magnetic fields, just as for S = 1/2 spin systems, because $g_{∥}$ is taken as positive in Equation (1). A more thorough treatment shows that the sign of the g-tensor sig’’nature, sign[Det(g)]>0, determines the counterclockwise (CCW) or clockwise (CW) direction of spin precession around an external magnetic field; see the literature [19,22,27] for details. In the case of an axially symmetric g-tensor, the two conditions are equivalent. 

However, the situation is somewhat different for the two interdoublet single-quantum FIR transitions, because the relation between the energy and m differs between these two allowed transitions. For the m=−3/2↔m=−1/2 transition, energy increases with m as for the EPR transition; but in the m=+1/2↔m=+3/2 transition, the energy decreases with  m. As we will show, they can only be excited by FIR radiation (its alternating magnetic field) with opposite circular polarizations.

### 3.2. Excitation by Circularly-Polarized Radiation

We use this differential response to FIR polarization for selective switching of SMMs. It allows strict selection of which transition is excited, based on the polarization of the FIR radiation. Even if the magnetic FIR transitions overlap or if the FIR radiation is not monochromatic, selective manipulation of the magnetization is possible using circularly-polarized FIR radiation.

We now examine an SMM driven by a circularly-polarized magnetic field perpendicular to Z at frequency ω. To simplify the discussion, we assume that radiation propagates along the positive direction of the external magnetic field, which is also the positive Z axis of our right-handed coordinate systems. Consequently, the spin magnetic moment movement and the electric (or magnetic) field of the radiation will rotate from the X axis toward the Y axis for right-handed circular polarization, but from the X axis toward the −Y axis for left-handed circular polarization (see Figure S1). The (rotating frame) spin Hamiltonian, ${\hat{H}}_{RF}$, in a frame rotating at ω is

(3) $${\hat{H}}_{RF}=D\left({\hat{S}}_{Z}^{2}-S\left(S+1\right)/3\right)+\left(\omega_{0}-\omega \right){\hat{S}}_{Z}+\omega_{1}{\hat{S}}_{X}.$$

Here, $\omega_{1}$ is the strength of the circularly-polarized magnetic field, $B_{1}$,

(4) $$\omega_{1}=g_{⊥}\beta B_{1}/\hbar .$$

The right-handed circular polarization applies to the rotating frame and $B_{1}$ when ω>0 and left-handed when ω<0. The typical field strengths are

(5) $$0<\omega_{1}\ll \omega_{0}\ll -D,$$

where $\omega \approx \omega_{0}$ for the EPR transition, or |ω|≈2|D| for the FIR interdoublet transitions. The spin Hamiltonians in the lab and rotating frames, shown in Equations (1) and (3), respectively, have the same eigenfunctions when $\omega_{1}\to 0$. The rotating frame eigenvalues are

(6) $$\omega_{m}=D\left(m^{2}-5/4\right)+\left(\omega_{0}-\omega \right)m.$$

The $\omega_{1}{\hat{S}}_{X}$ term in Equation (3) induces interdoublet transitions between states with Δm=±1 that are nearly degenerate in the rotating frame, which occurs at

(7) $$\omega \approx \omega_{m↔m-1}\left(=\omega_{m}-\omega_{m-1}\right)=\omega_{0}+D\left(2m-1\right)=\omega_{0}\mp 2D,$$

so that $\left|\omega -\omega_{m↔m-1}\right|<\omega_{1}$.

Within the upper Kramers doublet, $\omega_{1/2↔-1/2}=\omega_{0}>0$, and for one interdoublet transition, $\omega_{-1/2↔-3/2}=\omega_{0}-2D>0$, so that these transitions both require right-handed circularly-polarized magnetic fields, because ω>0. The other interdoublet FIR transition has $\omega_{+3/2↔+1/2}=\omega_{0}+2D<0$ and its negative resonance frequency requires a left-handed circularly-polarized field. Linearly-polarized fields have two oppositely rotating components and could excite any single quantum transition. So, circularly-polarized radiation provides additional selectivity for manipulation of the system states by suppressing unwanted transitions.

The circularly polarized radiation induces transitions in both directions between the two states involved (which is emphasized by the double arrow in the corresponding equations, for example, $\omega_{-1/2↔-3/2}$). Thus, low power, circularly polarized radiation applied for a long time will not coherently transfer the population from one state to another, but rather will incoherently equalize the populations of these two states by the saturation effect. Thus, there is a need for pulsed techniques for coherently switching the SMM states.

### 3.3. Manipulation of SMM System States

**Figure 2:**
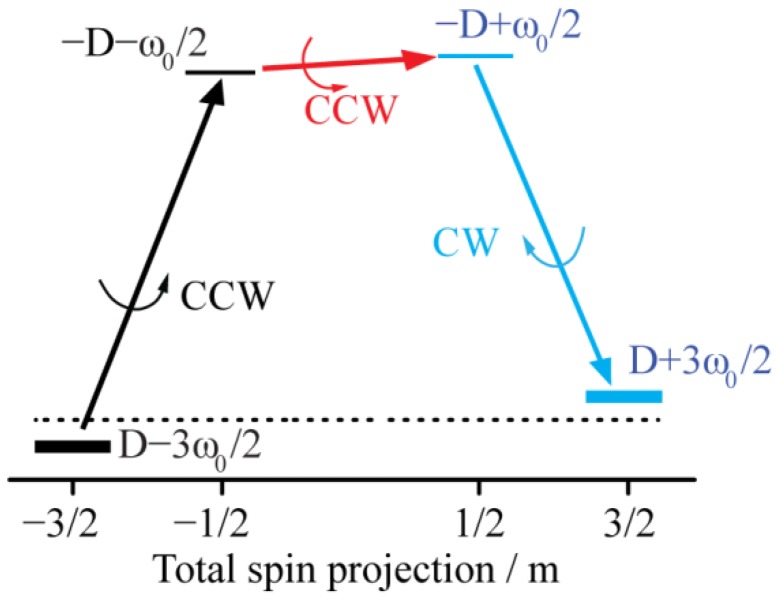
Schematic representation of switching between single molecular magnet (SMM) states. Three pulses resonant with three different transitions can switch the m=−3/2 SMM state to the m=+3/2 state. Horizontal lines denote energy levels of the *S* = 3/2 spin system in the presence of the magnetic field. Labels show the state energies and quantum numbers. Black and blue colors are used for negative and positive values of m, respectively. Straight arrows show the population transfer pathway, black and blue for the two far infrared (FIR) transitions, and red for the EPR transition. The circular-polarization direction is indicated by curved arrows, where CCW (counter clockwise, or right-handed circular polarization) and CW (clockwise, or left-handed circular polarization) refer to the rotation of the magnetic moment or field when viewed from the +Z direction.

Let us consider an SMM initially with its m=−3/2 state populated; shown in Figure 2. A right-handed, circularly-polarized π-pulse at the FIR frequency $\omega_{-1/2↔-3/2}=\omega_{0}-2D$ will exchange the populations and quantitatively transfer the population to the m=−1/2 state. That population can be transported further to the m=+1/2 state by a right-handed, circularly-polarized π-pulse at the mw frequency of $\omega_{1/2↔-1/2}=\omega_{0}$. Then, a left-handed, circularly-polarized π-pulse at the FIR frequency of $\omega_{3/2↔1/2}=\omega_{0}+2D$ can transfer that population to the m=3/2 state. This three-stage operation selectively switches the SMM state from m=−S to m=+S. The SMM state can be switched from m=+3/2 to m=−3/2 by the same pulses, but applied in the opposite order.

The details of the spin dynamics and of the evolution of the system density matrix under the applied pulse sequence are considered more thoroughly in the Supporting Information (SI). There, the interaction representation is used for the calculation of the propagators. It has an advantage over the traditional rotating frame presentation because it excludes the fast evolution of the density matrix for high spin systems. The SI also contains analytical formulae for the mean system Hamiltonian to first order in the small parameter $x=\omega_{1}/D$ and its zero order propagators. It is demonstrated that, for |x| < 10^−3^, the zero order propagators describe with good accuracy the evolution of the system populations during pulsed, circularly polarized fields. 

A noteworthy feature of this sequence of switching pulses is that it is not bidirectional. The first sequence described above quantitatively switches the SMM from m=−3/2 to m=+3/2, but does not quantitatively switch an SMM from m=+3/2 to m=−3/2. Another important feature is that the switching can be done quite rapidly in a time comparable to the combined lengths of the three π-pulses—perhaps a few nanoseconds—much faster than the magnetization relaxation times of a reasonably good SMM. It is shown in SI that the pulse sequence within a good accuracy produces a transmutation of level populations. Thus, the vector of populations $\left\{n_{-3/2}=1,n_{-1/2}=0,n_{+1/2}=0,n_{+3/2}=0\right\}$, with $n_{m}$ being the population of the m-th system level, will become {0,0,0,1}. The density matrix calculations show that quite small coherences will also be generated by the pulse sequence, which are not important for SMM applications, but, in principle, could be monitored by the pulse spectrometer, for example, to control the process of switching. The same pulses applied in the opposite order perform the reverse transmutation, $\left\{n_{-3/2}=0,n_{-1/2}=0,n_{+1/2}=0,n_{+3/2}=1\right\}\to \left\{1,0,0,0\right\}$. For quantitative switching, the initial state of the system must be quite pure; only one level should be populated, that with m=−3/2 in the first case and that with m=+3/2 in the second.

**Figure 3:**
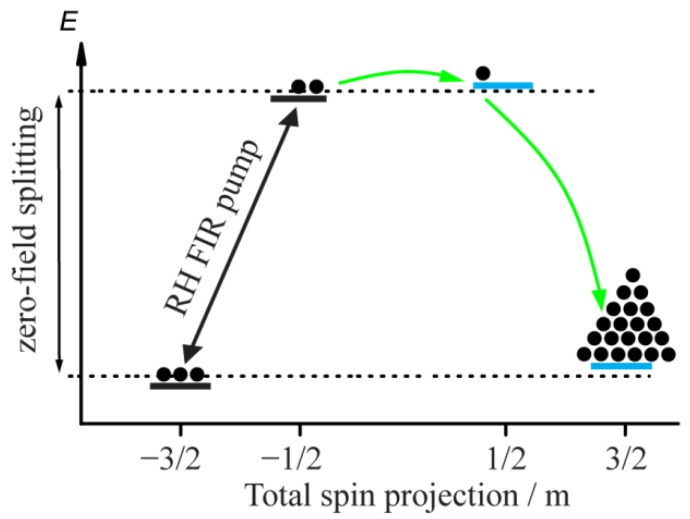
Schematic representation of switching between SMM states using right-handed circularly-polarized FIR radiation. Energy levels of the model system in an applied magnetic field are shown. Black and blue horizontal lines represent levels with negative and positive m, respectively. Black circles reflect the relative population of the energy levels. The black arrow denotes the spin transitions induced by right-handed circularly-polarized FIR radiation (RH FIR pump). The green arrows show the spin transitions induced by relaxation processes and leading to SMM switching.

Although the switching with circularly-polarized pulses can provide rapid and precise control of the SMM, circularly-polarized radiation can be used in less demanding ways. For example, the SMM can also be switched with a single quasi-continuous, circularly-polarized FIR frequency; shown in Figure 3. Pumping the SMM with prolonged right-handed circularly-polarized FIR radiation is sufficient to switch it into the m=+3/2 state utilizing spin relaxation processes (similar to what was done in the work of [16]). The population of the m=−3/2 state will gradually be excited into the m=−1/2 state and will relax directly back, or in two steps via the m=+1/2 state to the m=+3/2 state, where it will accumulate until the m=−3/2 state has been totally depleted. This could be useful, for example, to initialize a block of SMM memory in a desired state on the timescale of the spin-lattice relaxation of the upper Kramers doublet.

### 3.4. Influence of System Imperfections

#### 3.4.1. Non-Axial *g*-Tensor

In the case when the g-tensor is not axial, the effective ac magnetic field [27] will be elliptically polarized [19]. This means there will be two counter-rotating circularly polarized effective fields affecting the SMM spin, produced by the single circularly polarized radiation. The intensity of the unwanted counter-rotating component of the effective field would be proportional to the difference between the two in-plane principal values of the g-tensor, $\left(g_{XX}-g_{YY}\right)/2$, whereas the properly rotating effective field component will have an amplitude proportional to their mean value, $\left(g_{XX}+g_{YY}\right)/2$. Here, the X and Y are directions of the g-tenor principal axes with the system oriented so that the external static magnetic field is directed along the Z principal axis of the g-tensor. As a result, a rotating field at a frequency of ω~−2D>0 will induce −1/2↔−3/2 transitions with a relative intensity proportional to $\left(g_{XX}+g_{YY}\right)/2$. It will also induce +1/2↔+3/2 transitions with a relative intensity proportional to $\left(g_{XX}-g_{YY}\right)/2$, or a ratio $\left|\left(g_{XX}-g_{YY}\right)/\left(g_{XX}+g_{YY}\right)\right|$ times smaller than the desired ones. The effect of non-axial g-tensor can be mitigated to some degree by readjusting the circular polarizer to introduce an elliptically polarized component counteracting the unwanted counter-rotating effective field from the non-axial g-tensor.

For the 1/2↔−1/2 transition, the counter-rotating component does not have a direct effect on the population transfer, but may cause a small resonance frequency shift of the order of ${\left\{\omega_{1}\left(g_{XX}-g_{YY}\right)\right\}}^{2}/\omega_{0}$ owing to the Bloch–Siegert effect [31], which is easily compensated by a small mw frequency adjustment.

#### 3.4.2. Non-Axial ZFS

A non-axial ZFS has the strongest impact on the proposed unidirectional SMM switching. Firstly, it decreases the magnetic moments of the ground states because of an admixture of states with smaller |*m*|. Thus, for the *S* = 3/2 spin, the ground Kramers doublet is [25] $\left|\Psi_{\pm }\rangle =a|m=\pm 3/2\rangle +b|m=\mp 1/2\right\rangle$ (with external magnetic field directed along the Z axis). For these states, the mean values of spin projections, ${\bar{m}}_{\pm }$, are ${\bar{m}}_{\pm }=\pm {\left|a\right|}^{2}3/2\mp {\left|b\right|}^{2}/2$, where a and b are known functions of the ratio E/D (their exact forms are of no importance here), and ${\left|a\right|}^{2}+{\left|b\right|}^{2}=1$, ${\bar{m}}_{\pm }=\pm 3/2\mp 2{\left|b\right|}^{2}$. This means that $\left|{\bar{m}}_{\pm }\right|<3/2$ if b≠0, for the strong mixing case $b=\pm 1/\sqrt{2}$, so finally, $1/2\leq \left|{\bar{m}}_{\pm }\right|\leq 3/2$ for arbitrary ZFS asymmetry. This reduces the moment or strength of the SMM, making systems with non-axial ZFS less attractive for SMM applications. However, the most important impact of a non-axial ZFS is that the two ground levels of the SMM become coupled because the operator ${\hat{S}}_{X}$ slightly mixes these SMM levels, $\langle \Psi_{-}|{\hat{S}}_{X}|\Psi_{+}\rangle \sim {\left|b\right|}^{2}$, thus allowing a drastic enhancement of spin-lattice paramagnetic relaxation by thermal fluctuations of the local magnetic fields, and even by electrical field fluctuations that modulate the a and b coefficients. However, these are problems in the choice to use such non-axial SMMs and do not directly affect the utility of switching them using circularly polarized radiation.

#### 3.4.3. Inhomogeneous Broadening of the Resonance Lines

Inhomogeneous broadening of the FIR and EPR lines can be caused by interactions linear in the electron spin operator, for example, *g*-strain, with the same effects as for a system with an effective spin of 1/2. A transition will be excited when $\omega_{1}>\Delta \omega$, where Δω is the shift of the transition from the excitation frequency. If inhomogeneous broadening is strong, only a fraction of the spins will be excited, on the order of $\omega_{1}/\Delta \omega$. More accurate treatments for this case are readily available [19]. This means that either SMMs with rather sharp lines or powerful excitation pulses are required for SMM switching. 

The ZFS parameter D results from the static local electric field at the SMM, which may also have significant random strains. This results directly in inhomogeneous broadening and overlap of the +1/2↔+3/2 and −3/2↔−1/2 FIR transitions. Yet, the additional selectivity provided by the use of circularly polarized radiation makes it possible to selectively excite the +1/2↔+3/2 or the −3/2↔−1/2 FIR transitions, even when they overlap. This makes the ZeFIR scheme for selective switching of SMMs practical even for SMMs located on surfaces or embedded in inhomogeneous or doped materials. 

#### 3.4.4. Bloch–Siegert-Like Shifts

In the SI, the mean Hamiltonians are calculated for π-pulses at frequencies of the three resonance transitions of an SMM with corrections to the order of $\omega_{1}^{2}/D$. Some of these corrections are on the main diagonal of the spin Hamiltonian and present a first order energy shift during a pulse of radiation. Such shifts are analogs of the Bloch–Siegert shift of the resonance frequency in the rotating frame [31]. Let us consider the spin Hamiltonian, ${\hat{H}}_{F}$, for the fictitious spin F=1/2 corresponding to the +1/2↔+3/2 transition at frequency $\omega =\omega_{+3/2↔+1/2}=\omega_{0}+2D$, in the upper-left 2 × 2 block of the whole operator (S15) in the SI, 

(8) $${\hat{H}}_{F}=\left(\begin{array}{cc} 0 & \sqrt{3}\omega_{1}/2\\ \sqrt{3}\omega_{1}/2 & -\omega_{1}^{2}/2D \end{array}\right)=-\frac{\omega_{1}^{2}}{4D}\hat{1}+\frac{\omega_{1}^{2}}{2D}{\hat{F}}_{Z}+\sqrt{3}\omega_{1}{\hat{F}}_{X}.$$

Here, $\hat{1}$ is unity operator and may be omitted because it does not influence the dynamics of the fictitious spin, and ${\hat{F}}_{Q}$ are operators of the spin projection onto the axis Q (Q=X, Z). The factor $\omega_{1}^{2}/2D$ multiplying the operator ${\hat{F}}_{Z}$ describes the resonance line shift in the interaction representation frame. The analogous situation occurs for the other FIR transition, but its shift has the opposite sign, and the EPR transition has no such shift. These shifts are readily accommodated by a simple adjustment of the FIR excitation frequency. 

## 4. Conclusions

Circularly polarized radiation is able to provide precise and selective manipulation of the states of single molecular magnets of d-group Kramers ions with strong zero-field splitting. The ZeFIR approach proposed here enables unidirectional switching between the +*S* and −*S* states of *S* = 3/2 SMMs and could be extended to SMMs with larger *S*.

Circularly-polarized FIR fields also may be used to populate a specific state of the upper Kramers doublet, which is unpopulated when kT≪2|D|ℏ. For example, left-handed circularly-polarized FIR radiation inducing transitions between the m=+3/2 and m=+1/2 states will transiently populate the m=+1/2 state, and thus will create hyperpolarization of the upper Kramers doublet. This effect can be used to enhance the sensitivity of EPR measurements of the upper Kramers doublet or to prepare pure spin states, for example, for quantum information processing. 

## Supplementary Materials

The following are available online at https://www.mdpi.com/1996-1944/12/23/3865/s1, Part A: System Hamiltonian, circularly polarized magnetic field, and mean Hamiltonian in the interaction representation, Part B: System evolution under the action of π-pulses to zero order in *x* = *ω*_1_/*D* and an estimation of the first order corrections, Part C: Geometry scheme supposed for far infrared SMM irradiation.
