# Supplementary material for: Theoretical Basis for Switching a Kramers Single Molecular Magnet by Circularly-Polarized Radiation
Source: Materials (Basel). 2019 Nov 22;12(23):3865. doi: 10.3390/ma12233865 (PMC6926751; doi:10.3390/ma12233865)
Supplement: Supplementary file 1 [file materials-12-03865-s001.pdf]

materials

MDPI

Supporting Information

Theoretical Basis for Switching a Kramers Single Molecular Magnet by Circularly-Polarized Radiation

Alexander G. Maryasov [1,*], Michael K. Bowman [2,3], Matvey V. Fedin [4,5], and Sergey L. Veber [4,5,*]

Contents

Part A: System Hamiltonian, Circularly Polarized Magnetic Field, and Mean Hamiltonian in the Interaction Representation 1
Part B: System Evolution under the Action of $\boldsymbol{\pi}$-Pulses to Zero Order in $\boldsymbol{\omega 1 D}$ and an Estimation of the First Order Corrections 7
1. First order corrections. Case $x = -10 - 4$ 9
2. First order corrections. Case $x = -10 - 3$ 9
Part C: Geometry Scheme Supposed for Far Infrared SMM Irradiation 10
References 11

Part A: System Hamiltonian, Circularly Polarized Magnetic Field, and Mean Hamiltonian in the Interaction Representation

To describe the system dynamics under the action of an alternating magnetic field of far infrared (FIR) and microwave (mw) radiation, we use the density operator (sometimes called the density matrix) formalism widely used in both magnetic resonance and coherent optics [1]. The combination of two different ac magnetic field bands is a kind of double resonance technique, Zeeman–far infrared (ZeFIR) double resonance. The system energy levels of the SMM prototype are described by its spin Hamiltonian. The ideal prototype should have an axially symmetric spin Hamiltonian; in the lab frame, it is

$$\widehat{H}_0 = D\left(\hat{S}_Z^2 - S(S+1)/3\right) + \omega_0 \hat{S}_z. \quad \text{(S1)}$$

Here, D is the zero field splitting (ZFS) parameter and ω_0 is the Zeeman frequency; both parameters (and all energies below) are in angular frequency units, $1/s = 2\pi\ Hz$. Z is the direction of the axial axis of the ZFS and z is the quantization axis of the electron spin provided by the Zeeman interaction. The axially symmetry of the spin Hamiltonian means that the electric field caused by the central ion and ligands has high symmetry, not lower than C_{3v}. We consider a model system of a single molecular magnet (SMM) having electron spin $S = 3/2$. The zero field splitting Hamiltonian is diagonal in the molecular frame, where the system symmetry axis is chosen as the quantization axis. In the situation of interest, where we want to switch the SMM between its $+S$ and $-S$ states, the external magnetic field is chosen parallel to the molecular symmetry axis, so that the molecular and lab frames coincide. In the matrix form,

$$\widehat{H}_0 =$$

$$\begin{pmatrix} D + 3\,\omega_0/2 & 0 & 0 & 0 \\ 0 & -D + \omega_0/2 & 0 & 0 \\ 0 & 0 & -D - \omega_0/2 & 0 \\ 0 & 0 & 0 & D - 3\,\omega_0/2 \end{pmatrix} \quad \text{(S1.M)}$$

The interaction of the exciting circularly polarized radiation with the system is described in the lab frame as

$$\hat{H}_{ac,LF} = \omega_1\{\hat{S}_+ \exp(-i\omega t - i\alpha_j) + \hat{S}_- \exp(i\omega t + i\alpha_j)\}. \tag{S2}$$

Here, ω_1 is the amplitude of the oscillating ac field in angular frequency units defined in Equation (4), ω is the angular frequency of the circularly polarized ac magnetic field, and α_j is phase of the j-th pulse of the pulse sequence. The ladder operators, $\hat{S}_+$ and $\hat{S}_-$, are defined as usual, $\hat{S}_\pm = \hat{S}_x \pm i\hat{S}_y$. We assign the phase of the first pulse as zero, $\alpha_1 = 0$. The phases of the remaining pulses are relative to the phase of the first pulse. We continue to use the same convention in the main article to describe the handedness. The system Hamiltonian with circularly polarized ac field when the lab and molecular frames coincide is

$$\hat{H}_{LF} = \hat{H}_0 + \hat{H}_{ac,LF} =$$

$$\begin{pmatrix} D + 3\omega_0/2 & \sqrt{3}\,\omega_1 \exp[-i(\omega t + \alpha_j)]/2 & 0 & 0 \\ \sqrt{3}\,\omega_1 \exp[i(\omega t + \alpha_j)]/2 & -D + \omega_0/2 & \omega_1 \exp[-i(\omega t + \alpha_j)] & 0 \\ 0 & \omega_1 \exp[i(\omega t + \alpha_j)] & -D - \omega_0/2 & \sqrt{3}\,\omega_1 \exp[-i(\omega t + [illegible] \\ 0 & 0 & \sqrt{3}\,\omega_1 \exp[i(\omega t + \alpha_j)]/2 & D - 3\omega_0/2 \end{pmatrix} \tag{S3}$$

It is well known that π-pulses of a resonant ac magnetic field can invert the polarization of a pair of levels (in other words, such a pulse exchanges or transmutes the populations of the two levels), regardless of the phase of the pulse [2]. Equation (S3) allows us to calculate the respective spin dynamics. Calculations become simple if the Hamiltonian is made to be time-independent. The traditional way to remove the time dependence from the above Hamiltonian is to use a rotating frame (RF), which rotates in the same direction as the circularly polarized ac field. In this frame, (S3) becomes

$$\hat{H}_{RF} =$$

$$\begin{pmatrix} D + 3(\omega_0 - \omega)/2 & \sqrt{3}\,\omega_1 \exp[-i\alpha_j]/2 & 0 & 0 \\ \sqrt{3}\,\omega_1 \exp[i\alpha_j]/2 & -D + (\omega_0 - \omega)/2 & \omega_1 \exp[-i\alpha_j] & 0 \\ 0 & \omega_1 \exp[i\alpha_j] & -D - (\omega_0 - \omega)/2 & \sqrt{3}\,\omega_1 \exp[-i\alpha_j]/2 \\ 0 & 0 & \sqrt{3}\,\omega_1 \exp[i\alpha_j]/2 & D - 3(\omega_0 - \omega)/2 \end{pmatrix} \tag{S4}$$

Surprisingly, even for the EPR transition between the upper Kramers doublet sublevels, $m = -1/2$ and $m = +1/2$, when

$$\omega = \omega_0, \tag{S5}$$

the nutation or Rabi frequency for this transition is not always obvious. The effective Hamiltonian of the two states in resonance in this case is

$$\hat{H}_{2,3} = \begin{pmatrix} -D & \omega_1 exp(-i\alpha_2) \\ \omega_1 exp(i\alpha_2) & -D \end{pmatrix}. \tag{S6}$$

Here, $j = 2$ is used because the second pulse of our sequence (see the main text) has the frequency (S5) with phase α_2. The existence of the diagonal elements means that one more transformation is needed to exclude D and to get slow dynamics at the rate of the nutation or Rabi frequency. That transformation is not challenging for Equation (S6), but it is for the full Equation (S4). The transformation to the rotating frame is only sufficient for spin ½ systems.

There is a convenient way to exclude all terms other than those proportional to the Rabi frequency. The interaction representation (IR), which excludes the Hamiltonian $\hat{H}_0$ and transforms $\hat{H}_{ac,LF}$, is the most suitable technique to leave just the Rabi frequency as the driving force of the system during the oscillating field pulse. The density matrix, ρ_{IR}, and the system Hamiltonian, $\hat{H}_{IR}$, in the interaction representation are

$$\rho_{IR} = \exp\{i\hat{H}_0 t\}\,\rho_{LF}\,\exp\{-i\hat{H}_0 t\}, \tag{S7}$$

$$\hat{H}_{IR}(t) = \exp\{i\hat{H}_0 t\}\,\hat{H}_{ac,LF}\,\exp\{-i\hat{H}_0 t\}. \tag{S8}$$

Here, ρ_{LF} is the system density matrix in the lab frame and $\widehat{H}_{ac,LF}$ is the circular polarized field in the lab frame; shown in Equation (S2). The system dynamics in the interaction representation under the action of the oscillating field is described by the following equation:

$$\frac{d}{dt}\rho_{IR} = -i\left[\widehat{H}_{IR}(t), \rho_{IR}\right]. \tag{S9}$$

The advantage of the interaction representation as compared with the rotating frame is that, in the absence of the oscillating field, with $\omega_1 = 0$, so that $\widehat{H}_{IR} = 0$, the system does not evolve, and ρ_{IR} is constant. This means that only propagators for the pulses need to be calculated in the interaction representation, whereas non-trivial free evolution takes place between pulses in the rotating frame.

We are interested in calculations of the evolution of populations under the action of a sequence of π-pulses exchanging populations of the two resonant sublevels (for each of three single quantum transitions in our S = 3/2 case). If ρ_{LF} is diagonal in the basis of the system eigenstates, then $\rho_{IR} = \rho_{LF}$. The fact that $\exp\{i\widehat{H}_0 t\}$ and $\exp\{-i\widehat{H}_0 t\}$ are also diagonal in that basis proves that this equality is true; see Equation (S7). This means that it will be enough to find the diagonal part of ρ_{IR} after a pulse sequence as far as SMM switching is concerned. Off-diagonal elements of density operator may be calculated and measured experimentally to check the accuracy of tuning of ZeFIR pulses.

The operator $\widehat{H}_{ac,LF}$ (S2) is a sum of two ladder operators and the exponential operators in (S8), $\exp\{\pm i\widehat{H}_0 t\}$, are diagonal in the basis chosen, thus making calculations trivial, so that (S8) becomes

$$\widehat{H}_{IR}(t) =$$

$$\begin{pmatrix} 0 & \frac{\sqrt{3}}{2}\omega_1 \exp(-i(\alpha_j + t(-2D + \omega - \omega_0))) & 0 & 0 \\ \frac{\sqrt{3}}{2}\omega_1 \exp(i(\alpha_j + t(-2D + \omega - \omega_0))) & 0 & \omega_1 \exp(-i(\alpha_j + t(\omega - \omega_0))) & 0 \\ 0 & \omega_1 \exp(i(\alpha_j + t(\omega - \omega_0))) & 0 & \frac{\sqrt{3}}{2}\omega_1 \exp(-i(\alpha_j + t(2D + \omega - \omega_0)) \\ 0 & 0 & \frac{\sqrt{3}}{2}\omega_1 \exp(i(\alpha_j + t(2D + \omega - \omega_0))) & 0 \end{pmatrix} \tag{S10}$$

In contrast to Equation (S4), the non-zero terms in the above Hamiltonian simply induce transitions between the system eigenstates. These transitions are most effective when the ac frequency ω is in resonance with the energy difference in the lab frame. In the interaction representation, the coupling terms become constant and do not oscillate, producing evolution at a constant Rabi frequency.

Let us consider the evolution of the system during a resonant pulse. First, we consider the $+3/2 \leftrightarrow +1/2$ resonance, where $\omega = \omega_0 + 2D$, so that (S10) becomes

$$\widehat{H}_{+3/2\leftrightarrow+1/2}(t) =$$

$$\begin{pmatrix} 0 & \sqrt{3}\,\omega_1/2 & 0 & 0 \\ \sqrt{3}\,\omega_1/2 & 0 & \omega_1 \exp\{-i\,2Dt\} & 0 \\ 0 & \omega_1 \exp\{i\,2Dt\} & 0 & \sqrt{3}\,\omega_1/2 \exp\{-i\,4Dt\} \\ 0 & 0 & \sqrt{3}\,\omega_1/2 \exp\{i\,4Dt\} & 0 \end{pmatrix} \tag{S11}$$

Without loss of generality, we can take $\alpha_1 = 0$. To calculate the system evolution under the action of a resonant pulse with duration t_p, it is suitable to use the mean Hamiltonian theory in the form of the Magnus expansion [3], which is definitely applicable in this situation.

The first term of the Magnus expansion averages the above Hamiltonian over the pulse length,

$$\overline{\widehat{H}}^{(1)}_{+3/2\leftrightarrow+1/2} = \int_0^{t_p} \widehat{H}_{+3/2\leftrightarrow+1/2}(t)dt/t_p = \tag{S12}$$

$$\begin{pmatrix} 0 & \frac{\sqrt{3}\,\omega_1}{2} & 0 & 0 \\ \frac{\sqrt{3}\,\omega_1}{2} & 0 & \frac{i\,\omega_1^2\left(e^{-i\,2\,D\,t_p}-1\right)}{2\,D\,(\omega_1\,t_p)} & 0 \\ 0 & -\frac{i\,\omega_1^2\left(e^{i\,2\,D\,t_p}-1\right)}{2\,D\,(\omega_1\,t_p)} & 0 & \frac{i\sqrt{3}\,\omega_1^2\left(e^{-i\,4\,D\,t_p}-1\right)}{8\,D\,(\omega_1\,t_p)} \\ 0 & 0 & -\frac{i\sqrt{3}\,\omega_1^2\left(e^{i\,4\,D\,t_p}-1\right)}{8\,D\,(\omega_1\,t_p)} & 0 \end{pmatrix}$$

Note that for this transition, the Rabi frequency is $\sqrt{3}\omega_1$ and for a π-pulse,

$$\omega_1 t_{p\pi} = \sqrt{3}\pi/3. \tag{S13}$$

This means that all corrections will be on the order of $\omega_1/|D|$. For the systems of interest, this parameter is quite small and does not exceed 10^{-4} – 10^{-3}. It should be noted here that, for the case of continuous wave (cw) spectroscopy, the time averaging period t_p tends to infinity, thus allowing omission of Hamiltonian elements having t_p in a denominator. The difference between cw and pulse spectroscopies cases is that corrections of the first and of the second orders have different relative importance for cw, but the same for pulses; see below.

The second term of the Magnus expansion is

$$\bar{\hat{H}}^{(2)}_{+3/2\leftrightarrow+1/2} = -i\int_0^{t_p} dt_2 \int_0^{t_2} \left[\hat{H}(t_2), \hat{H}(t_1)\right] dt_1/2t_p =$$

$$\begin{pmatrix} 0 & 0 & \frac{\sqrt{3}\,\omega_1^2\,e^{-i\,D\,t_p}\left(\mathrm{Sinc}[D\,t_p]-\mathrm{Cos}[D\,t_p]\right)}{4\,D} & 0 \\ 0 & \frac{\omega_1^2\left(\mathrm{Sinc}[2\,D\,t_p]-1\right)}{2\,D} & 0 & \frac{\omega_1^2\,e^{-i\,3\,D\,t_p}\,\mathrm{Sin}[D\,t_p]^2\,\mathrm{Sinc}[D\,t_p]}{4\sqrt{3}\,D} \\ \frac{\sqrt{3}\,\omega_1^2\,e^{i\,D\,t_p}\left(\mathrm{Sinc}[D\,t_p]-\mathrm{Cos}[D\,t_p]\right)}{4\,D} & 0 & \frac{\omega_1^2\left(5-8\,\mathrm{Sinc}[2\,D\,t_p]+3\,\mathrm{Sinc}[4\,D\,t_p]\right)}{16\,D} & 0 \\ 0 & \frac{\omega_1^2\,e^{i\,3\,D\,t_p}\,\mathrm{Sin}[D\,t_p]^2\,\mathrm{Sinc}[D\,t_p]}{4\sqrt{3}\,D} & 0 & \frac{3\,\omega_1^2\left(1-\mathrm{Sinc}[4\,D\,t_p]\right)}{16\,D} \end{pmatrix} \tag{S14}$$

Here, $Sinc(x) = Sin(x)/x$. The comparison of the two terms of the Magnus expansion demonstrates that these are of the same order for a π-pulse. In our case, $|D|t_p = (\omega_1 t_p)(|D|/\omega_1) \gg 1$, so that $Sinc(Dt_p)$ provides an additional small factor of ω_1/D to each term containing it. Taking this into account, $Sinc(nDt_p)$ (for integer n) may be neglected relative to terms of the order of unity. Summing up the two terms of the Magnus series for the mean Hamiltonian, $\bar{\hat{H}}$, we get

$$\bar{\hat{H}}_{+3/2\leftrightarrow+1/2} \approx$$

$$\begin{pmatrix} 0 & \frac{\sqrt{3}\,\omega_1}{2} & \frac{-\sqrt{3}\,\omega_1^2\left(1+e^{-i\,2\,D\,t_p}\right)}{8\,D} & 0 \\ \frac{\sqrt{3}\,\omega_1}{2} & -\frac{\omega_1^2}{2\,D} & \frac{i\,\omega_1^2\left(e^{-i\,2\,D\,t_p}-1\right)}{2\,D\,(\omega_1\,t_p)} & 0 \\ \frac{-\sqrt{3}\,\omega_1^2\left(1+e^{i\,2\,D\,t_p}\right)}{8\,D} & -\frac{i\,\omega_1^2\left(e^{i\,2\,D\,t_p}-1\right)}{2\,D\,(\omega_1\,t_p)} & \frac{5\,\omega_1^2}{16\,D} & \frac{i\sqrt{3}\,\omega_1^2\left(e^{-i\,4\,D\,t_p}-1\right)}{8\,D\,(\omega_1\,t_p)} \\ 0 & 0 & -\frac{i\sqrt{3}\,\omega_1^2\left(e^{i\,4\,D\,t_p}-1\right)}{8\,D\,(\omega_1\,t_p)} & \frac{3\,\omega_1^2}{16\,D} \end{pmatrix} \tag{S15}$$

For the π-pulse, we use not the mean Hamiltonian itself, but the dimensionless quantity $\hat{\Phi}_{+3/2\leftrightarrow+1/2}(\pi) = \bar{\hat{H}}_{+3/2\leftrightarrow+1/2} t_{p\pi}$, which will be used below for propagator calculations,

$$\hat{\Phi}_{+3/2\leftrightarrow+1/2}(\pi) \approx$$

$$\begin{pmatrix} 0 & \frac{\pi}{2} & \frac{-\pi\,\omega_1\left(1+e^{-i\,2\,D\,t_p}\right)}{8\,D} & 0 \\ \frac{\pi}{2} & -\frac{\sqrt{3}\,\pi\,\omega_1}{6\,D} & \frac{i\,\omega_1\left(e^{-i\,2\,D\,t_p}-1\right)}{2\,D} & 0 \\ \frac{-\pi\,\omega_1\left(1+e^{i\,2\,D\,t_p}\right)}{8\,D} & -\frac{i\,\omega_1\left(e^{i\,2\,D\,t_p}-1\right)}{2\,D} & \frac{5\sqrt{3}\,\pi\,\omega_1}{48\,D} & \frac{i\sqrt{3}\,\omega_1\left(e^{-i\,4\,D\,t_p}-1\right)}{8\,D} \\ 0 & 0 & -\frac{i\sqrt{3}\,\omega_1\left(e^{i\,4\,D\,t_p}-1\right)}{8\,D} & \frac{\sqrt{3}\,\pi\,\omega_1}{16\,D} \end{pmatrix}$$

(S16)

For the resonance frequency coupling the second and the third levels, $\omega = \omega_0$, the interaction representation spin Hamiltonian is

$\hat{H}_{+1/2\leftrightarrow-1/2}(t)$=

$$\begin{pmatrix} 0 & \frac{\sqrt{3}}{2}\,\omega_1\exp\left(-i\left(\alpha_2-2\,D\,t\right)\right) & 0 & 0 \\ \frac{\sqrt{3}}{2}\,\omega_1\exp\left(i\left(\alpha_2-2\,D\,t\right)\right) & 0 & \omega_1\exp\left(-i\,\alpha_2\right) & 0 \\ 0 & \omega_1\exp\left(i\,\alpha_2\right) & 0 & \frac{\sqrt{3}}{2}\,\omega_1\exp\left(-i\left(\alpha_2+2\,D\,t\right)\right) \\ 0 & 0 & \frac{\sqrt{3}}{2}\,\omega_1\exp\left(i\left(\alpha_2+2\,D\,t\right)\right) & 0 \end{pmatrix}$$

(S17)

Using the same procedure as for the previous transition, and omitting all terms with $Sinc(nDt_p)$ functions, we get
$\bar{\bar{H}}_{+1/2\leftrightarrow-1/2} \approx$

$$\begin{pmatrix} \frac{3\,\omega_1^2}{8\,D} & \frac{i\sqrt{3}\,\omega_1^2\,e^{-i\,\alpha_2}\left(1-e^{i\,2\,D\,t_p}\right)}{4\,D\,(\omega_1\,t_p)} & -\frac{\sqrt{3}\,\omega_1^2\,e^{-2\,i\,\alpha_2}\left(1+e^{i\,2\,D\,t_p}\right)}{8\,D} & 0 \\ -\frac{i\sqrt{3}\,\omega_1^2\,e^{i\,\alpha_2}\left(1-e^{-i\,2\,D\,t_p}\right)}{4\,D\,(\omega_1\,t_p)} & -\frac{3\,\omega_1^2}{8\,D} & \omega_1\,e^{-i\,\alpha_2} & -\frac{\sqrt{3}\,\omega_1^2\,e^{-2\,i\,\alpha_2}\left(1+e^{-i\,2\,D\,t_p}\right)}{8\,D} \\ -\frac{\sqrt{3}\,\omega_1^2\,e^{2\,i\,\alpha_2}\left(1+e^{-i\,2\,D\,t_p}\right)}{8\,D} & \omega_1\,e^{i\,\alpha_2} & -\frac{3\,\omega_1^2}{8\,D} & -\frac{i\sqrt{3}\,\omega_1^2\,e^{-i\,\alpha_2}\left(1-e^{-i\,2\,D\,t_p}\right)}{4\,D\,(\omega_1\,t_p)} \\ 0 & -\frac{\sqrt{3}\,\omega_1^2\,e^{2\,i\,\alpha_2}\left(1+e^{i\,2\,D\,t_p}\right)}{8\,D} & \frac{i\sqrt{3}\,\omega_1^2\,e^{i\,\alpha_2}\left(1-e^{i\,2\,D\,t_p}\right)}{4\,D\,(\omega_1\,t_p)} & \frac{3\,\omega_1^2}{8\,D} \end{pmatrix}$$

(S18)

For this transition, the Rabi frequency is two times higher than for spin ½ so for a π-pulse

$$\omega_1 t_{p\pi} = \pi/2, \qquad \text{(S19)}$$

so the above Hamiltonian provides a phase operator in the form

$\hat{\Phi}_{+1/2\leftrightarrow-1/2}(\pi) = \bar{\bar{H}}_{+1/2\leftrightarrow-1/2}\,t_{p\pi} =$

$$\begin{pmatrix} \frac{3\pi\omega_1}{16D} & \frac{i\sqrt{3}\,\omega_1 e^{-i\alpha_2}\left(1-e^{i2Dt_p}\right)}{4D} & -\frac{\sqrt{3}\,\pi\omega_1 e^{-2i\alpha_2}\left(1+e^{i2Dt_p}\right)}{16D} & 0 \\ -\frac{i\sqrt{3}\,\omega_1 e^{i\alpha_2}\left(1-e^{-i2Dt_p}\right)}{4D} & -\frac{3\pi\omega_1}{16D} & \pi e^{-i\alpha_2}/2 & -\frac{\sqrt{3}\,\pi\omega_1 e^{-2i\alpha_2}\left(1+e^{-i2Dt_p}\right)}{16D} \\ -\frac{\sqrt{3}\,\pi\omega_1 e^{2i\alpha_2}\left(1+e^{-i2Dt_p}\right)}{16D} & \pi e^{i\alpha_2}/2 & -\frac{3\pi\omega_1}{16D} & -\frac{i\sqrt{3}\,\omega_1 e^{-i\alpha_2}\left(1-e^{-i2Dt_p}\right)}{4D} \\ 0 & -\frac{\sqrt{3}\,\pi\omega_1 e^{2i\alpha_2}\left(1+e^{i2Dt_p}\right)}{16D} & \frac{i\sqrt{3}\,\omega_1 e^{i\alpha_2}\left(1-e^{i2Dt_p}\right)}{4D} & \frac{3\pi\omega_1}{16D} \end{pmatrix} \tag{S20}$$

When the frequency is chosen to couple the third and the fourth levels, $\omega = \omega_0 - 2D$, the time-dependent IR spin Hamiltonian is

$\hat{H}_{-1/2\leftrightarrow-3/2}(t) =$

$$\begin{pmatrix} 0 & \frac{\sqrt{3}}{2}\omega_1 \exp\left(i\left(-\alpha_3+4Dt\right)\right) & 0 & 0 \\ \frac{\sqrt{3}}{2}\omega_1 \exp\left(i\left(\alpha_3-4Dt\right)\right) & 0 & \omega_1 \exp\left(i\left(-\alpha_3+2Dt\right)\right) & 0 \\ 0 & \omega_1 \exp\left(i\left(\alpha_3-2Dt\right)\right) & 0 & \frac{\sqrt{3}}{2}\omega_1 \exp\left(-i\alpha_3\right) \\ 0 & 0 & \frac{\sqrt{3}}{2}\omega_1 \exp\left(i\alpha_3\right) & 0 \end{pmatrix} \tag{S21}$$

The mean Hamiltonian obtained after the same set of operations is

$\bar{\hat{H}}_{-1/2\leftrightarrow-3/2} \approx$

$$\begin{pmatrix} \frac{3\omega_1^2}{16D} & \frac{i\sqrt{3}\,\omega_1^2 e^{-i\alpha_3}\left(1-e^{i4Dt_p}\right)}{8D\left(\omega_1 t_p\right)} & 0 & 0 \\ -\frac{i\sqrt{3}\,\omega_1^2 e^{i\alpha_3}\left(1-e^{-i4Dt_p}\right)}{8D\left(\omega_1 t_p\right)} & \frac{5\omega_1^2}{16D} & \frac{i\omega_1^2 e^{-i\alpha_3}\left(1-e^{i2Dt_p}\right)}{2D\left(\omega_1 t_p\right)} & -\frac{\sqrt{3}\,\omega_1^2 e^{-2i\alpha_3}\left(1+e^{i2Dt_p}\right)}{8D} \\ 0 & -\frac{i\omega_1^2 e^{i\alpha_3}\left(1-e^{-i2Dt_p}\right)}{2D\left(\omega_1 t_p\right)} & -\frac{\omega_1^2}{2D} & \frac{\sqrt{3}}{2}\omega_1 e^{-i\alpha_3} \\ 0 & -\frac{\sqrt{3}\,\omega_1^2 e^{2i\alpha_3}\left(1+e^{-i2Dt_p}\right)}{8D} & \frac{\sqrt{3}}{2}\omega_1 e^{i\alpha_3} & 0 \end{pmatrix} \tag{S22}$$

For this transition, for π-pulse, Equation (S13) is valid, so that

$\hat{\Phi}_{-1/2\leftrightarrow-3/2}(\pi) = \bar{\hat{H}}_{-1/2\leftrightarrow-3/2} t_{p\pi} =$

$$\begin{pmatrix} \frac{\sqrt{3}\,\pi\omega_1}{16D} & \frac{i\sqrt{3}\,\omega_1 e^{-i\alpha_3}\left(1-e^{i4Dt_p}\right)}{8D} & 0 & 0 \\ -\frac{i\sqrt{3}\,\omega_1 e^{i\alpha_3}\left(1-e^{-i4Dt_p}\right)}{8D} & \frac{5\sqrt{3}\,\pi\omega_1}{48D} & \frac{i\omega_1 e^{-i\alpha_3}\left(1-e^{i2Dt_p}\right)}{2D} & -\frac{\pi\omega_1 e^{-2i\alpha_3}\left(1+e^{i2Dt_p}\right)}{8D} \\ 0 & -\frac{i\omega_1 e^{i\alpha_3}\left(1-e^{-i2Dt_p}\right)}{2D} & -\frac{\sqrt{3}\,\pi\omega_1}{6D} & \pi e^{-i\alpha_3}/2 \\ 0 & -\frac{\pi\omega_1 e^{2i\alpha_3}\left(1+e^{-i2Dt_p}\right)}{8D} & \pi e^{i\alpha_3}/2 & 0 \end{pmatrix} \tag{S23}$$

Part B: System Evolution under the Action of π-Pulses to Zero Order in ω_1/D and an Estimation of the First Order Corrections

We consider here paramagnetic centers that may potentially serve as SMMs, thus having values of D in the THz range (~10^{13} 1/s), whereas pulse radiation sources can produce oscillating magnetic field with amplitudes up to 10^9 1/s (~150 MHz), providing the possibility of having π-pulses not shorter than a few nanoseconds. The estimate of $\omega_1/|D|\sim 10^{-4}$ allows the use of the zero order approximation for this parameter for the calculations of the system propagators for the sequences of π-pulses needed to switch the SMM from its $\pm S$ state to $\mp S$ state. Corrections to the density matrix owing to terms of the order of $\omega_1/|D|$ are estimated numerically later.

The propagator for the system evolution during a pulse coupling the m and $m-1$ system states is

$$U_{m,m-1}(t_p) = exp\left\{-i\bar{\bar{H}}_{m,m-1}t_p\right\}, \tag{S24}$$

and for a π-pulse, it is

$$U_{m,m-1}(\pi) = exp\left\{-i\hat{\Phi}_{\mathrm{m}\leftrightarrow m-1}(\pi)\right\}. \tag{S24.1}$$

In zero order, the mean Hamiltonians in the interaction representation for each transition of interest have only two non-zero single quantum transition off-diagonal elements. It is easy to construct the propagator for a Hamiltonian of this type, the two off-diagonal real non-zero elements may be treated as the operator $\hat{S}_X^F$ of some fictitious spin $S^F = 1/2$. Thus, we have zero-order propagators for the three transitions

$$U_{3/2,\,1/2}(t_p) = \begin{pmatrix} \cos\left(\frac{\pi t_p}{2 t_{p\pi}}\right) & -i\sin\left(\frac{\pi t_p}{2 t_{p\pi}}\right) & 0 & 0 \\ -i\sin\left(\frac{\pi t_p}{2 t_{p\pi}}\right) & \cos\left(\frac{\pi t_p}{2 t_{p\pi}}\right) & 0 & 0 \\ 0 & 0 & 1 & 0 \\ 0 & 0 & 0 & 1 \end{pmatrix} \tag{S25.1}$$

$$U_{1/2,\,-1/2}(t_p) = \begin{pmatrix} 1 & 0 & 0 & 0 \\ 0 & \cos\left(\frac{\pi t_p}{2 t_{p\pi}}\right) & -i\,e^{-i\alpha_2}\sin\left(\frac{\pi t_p}{2 t_{p\pi}}\right) & 0 \\ 0 & -i\,e^{i\alpha_2}\sin\left(\frac{\pi t_p}{2 t_{p\pi}}\right) & \cos\left(\frac{\pi t_p}{2 t_{p\pi}}\right) & 0 \\ 0 & 0 & 0 & 1 \end{pmatrix} \tag{S25.2}$$

$$U_{-1/2,\,-3/2}(t_p) = \begin{pmatrix} 1 & 0 & 0 & 0 \\ 0 & 1 & 0 & 0 \\ 0 & 0 & \cos\left(\frac{\pi t_p}{2 t_{p\pi}}\right) & -i\,e^{-i\alpha_3}\sin\left(\frac{\pi t_p}{2 t_{p\pi}}\right) \\ 0 & 0 & -i\,e^{i\alpha_3}\sin\left(\frac{\pi t_p}{2 t_{p\pi}}\right) & \cos\left(\frac{\pi t_p}{2 t_{p\pi}}\right) \end{pmatrix} \tag{S25.3}$$

For π-pulses for all the above propagators, we get

$U_{3/2,\,1/2}(\pi) =$

$$\begin{pmatrix} 0 & -i & 0 & 0 \\ -i & 0 & 0 & 0 \\ 0 & 0 & 1 & 0 \\ 0 & 0 & 0 & 1 \end{pmatrix} \tag{S26.1}$$

$U_{1/2,\,-1/2}(\pi) =$

$$\begin{pmatrix} 1 & 0 & 0 & 0 \\ 0 & 0 & -i\, e^{-i\,\alpha_2} & 0 \\ 0 & -i\, e^{i\,\alpha_2} & 0 & 0 \\ 0 & 0 & 0 & 1 \end{pmatrix} \tag{S26.2}$$

$U_{-1/2,\,-3/2}(\pi) =$

$$\begin{pmatrix} 1 & 0 & 0 & 0 \\ 0 & 1 & 0 & 0 \\ 0 & 0 & 0 & -i\, e^{-i\,\alpha_3} \\ 0 & 0 & -i\, e^{i\,\alpha_3} & 0 \end{pmatrix} \tag{S26.3}$$

Note that the π-pulse propagators (S26) perform permutations on the respective levels populations and, as expected, this permutation does not depend on the pulse phase α_j.

If the initial system state is given as a diagonal density operator in the lab frame, it is the same after transformation into the interaction representation for our system. Denoting the initial system state as ρ_0,

$$\rho_0 = DiagonalMatrix(n_1, n_2, n_3, n_4), \tag{S27}$$

after application of the resonance π-pulse between levels $3/2$, $1/2$, we get

$$\rho_1 = U_{3/2,\,1/2}(\pi)\rho_0 U^{-1}_{3/2,\,1/2}(\pi) = DiagonalMatrix(n_2, n_1, n_3, n_4). \tag{S28}$$

In the state ρ_1, after the pulse, the populations of the two first states do permute. The next permutation takes place after application of a resonant π-pulse between levels $1/2, -1/2$,

$$\rho_2 = U_{1/2,-1/2}(\pi)\rho_1 U^{-1}_{1/2,-1/2}(\pi) = DiagonalMatrix(n_2, n_3, n_1, n_4). \tag{S29}$$

Finally, after a resonant π-pulse between levels $-1/2, -3/2$, we get

$$\rho_3 = U_{-1/2,-3/2}(\pi)\rho_2 U^{-1}_{-1/2,-3/2}(\pi) = DiagonalMatrix(n_2, n_3, n_4, n_1). \tag{S30}$$

This means that after a three-pulse sequence, the system has changed the state (n_1, n_2, n_3, n_4) to the state (n_2, n_3, n_4, n_1). Thus, if the $m = +S = 3/2$ state was initially populated ($n_1 = 1$), then the final populated level is $m = -S = -3/2$, which means a switching of the SMM, $(1,0,0,0) \rightarrow (0,0,0,1)$. Applying the pulse sequence in the opposite order, the system state (n_1, n_2, n_3, n_4) will be changed to (n_4, n_1, n_2, n_3) with reverse switching taking place, $(0,0,0,1) \rightarrow (1,0,0,0)$. Let us review the phase independence of the switching; it takes place because each π-pulse rotation produces the same result, regardless of a particular direction of the resonant field in the interaction representation frame, it is enough that the plane where the circularly polarized field oscillates is perpendicular to the axial system axis.

Now, we consider the first order corrections in the small parameter $x = \omega_1/D$ for the π-pulse propagators (S24); this will be done numerically. It is possible to reduce the number of parameters by one by omitting elsewhere the fast oscillating terms $exp(\pm inDt_p)$ $(n = 2, 4)$, because imperfect pulses or even a rather small D-strain will average these out. Anyway, our estimates are performed to check the order of magnitude of distortions introduced by off-resonance effects. After neglecting the terms mentioned, the operators (S16), (S20), and (S23) depend only on the parameter x.

Let us calculate numerically propagators S(24.1) with higher accuracy than (S26) for $x = -10^{-4}$ and -10^{-3}, keeping in mind that the systems of interest have a negative value of D. We present here not the propagators themselves, but the results of the pulses. The system initial state is taken as

$$\rho_0 = DiagonalMatrix(1, 0, 0, 0), \tag{S31}$$

so that just the $m = +S = 3/2$ state is populated.

1. First order corrections. Case $x = -10^{-4}$

Firstly, we assume the pulses have synchronized phases, $\alpha_2 = \alpha_3 = 0$.

After action of the first resonance π-pulse the system density operator is

$\rho_1 = U_{3/2,\,1/2}(\pi)\rho_0 U^{-1}_{3/2,\,1/2}(\pi) =$

$$\begin{pmatrix} 8.33333\times10^{-10} & -0.0000288675 - 9.27935\times10^{-10}\,i & 1.97194\times10^{-10} & 0 \\ -0.0000288675 + 9.27935\times10^{-10}\,i & 1. & -6.83099\times10^{-6} - 1.16537\times10^{-9}\,i & 0 \\ 1.97194\times10^{-10} & -6.83099\times10^{-6} + 1.16537\times10^{-9}\,i & 0 & 0 \\ 0 & 0 & 0 & 0 \end{pmatrix}$$

(S32.1)

Populations are changed as in an ideal case, but also some off diagonal elements, coherences, are created. Amplitudes of those are less than $|x|$. Density operator after the second pulse is

$\rho_2 = U_{1/2,-1/2}(\pi)\rho_0 U^{-1}_{1/2,-1/2}(\pi) =$

$$\begin{pmatrix} 6.0972\times10^{-9} & 0. + 5.33395\times10^{-10}\,i & 7.36356\times10^{-9} - 0.0000780846\,i & 0. + 4.61934\times10^{-10}\,i \\ 0. - 5.33395\times10^{-10}\,i & 0 & -6.83099\times10^{-6} + 2.93285\times10^{-10}\,i & 0 \\ 7.36356\times10^{-9} + 0.0000780846\,i & -6.83099\times10^{-6} - 2.93285\times10^{-10}\,i & 1. & -5.91581\times10^{-6} - 6.85734\times10^{-10}\,i \\ 0. - 4.61934\times10^{-10}\,i & 0 & -5.91581\times10^{-6} + 6.85734\times10^{-10}\,i & 0 \end{pmatrix}$$

(S32.2)

Finally, after the third pulse, we get

$\rho_3 = U_{-1/2,-3/2}(\pi)\rho_0 U^{-1}_{-1/2,-3/2}(\pi) =$

$$\begin{pmatrix} 6.0972\times10^{-9} & 0. + 4.97102\times10^{-9}\,i & 1.79217\times10^{-9} & 0.0000780846 + 1.43432\times10^{-8}\,i \\ 0. - 4.97102\times10^{-9}\,i & 4.05285\times10^{-9} & 0. - 1.46115\times10^{-9}\,i & 4.21011\times10^{-9} - 0.000063662\,i \\ 1.79217\times10^{-9} & 0. + 1.46115\times10^{-9}\,i & 5.26781\times10^{-10} & 0.0000229517 - 6.77076\times10^{-10}\,i \\ 0.0000780846 - 1.43432\times10^{-8}\,i & 4.21011\times10^{-9} + 0.000063662\,i & 0.0000229517 + 6.77076\times10^{-10}\,i & 1. \end{pmatrix}$$

(S32.3)

The pulse sequence works perfectly, populations are distorted at the level of numerical accuracy, and amplitudes of coherences are about $|x| = 10^{-4}$ or less. There is no purpose to vary the phases of the two last pulses; these would redistribute amplitudes of coherences that are rather small, these quantities are considered below for higher field strengths.

2. First order corrections. Case $x = -10^{-3}$

Again, synchronized phases of $\alpha_2 = \alpha_3 = 0$ are used initially. In the case of a ten-fold stronger oscillating magnetic field (and ten times shorter pulses), we get density operators ρ_1, ρ_2, ρ_3, shown below:

$$\begin{pmatrix} 8.33333\times10^{-8} & -0.000288675-9.27935\times10^{-8}\,i & 1.97194\times10^{-8} & 0 \\ -0.000288675+9.27935\times10^{-8}\,i & 1. & -0.00006831-1.16537\times10^{-7}\,i & -9.41529\times10^{-9} \\ 1.97194\times10^{-8} & -0.00006831+1.16537\times10^{-7}\,i & 4.66626\times10^{-9} & 0 \\ 0 & -9.41529\times10^{-9} & 0 & 0 \end{pmatrix}$$

$$\begin{pmatrix} 6.0972\times10^{-7} & 0.+5.33394\times10^{-8}\,i & 7.36356\times10^{-7}-0.000780845\,i & 0.+4.61932\times10^{-8}\,i \\ 0.-5.33394\times10^{-8}\,i & 4.66624\times10^{-9} & -0.0000683099+2.93285\times10^{-8}\,i & 4.04108\times10^{-9} \\ 7.36356\times10^{-7}+0.000780845\,i & -0.0000683099-2.93285\times10^{-8}\,i & 0.999999 & -0.000059158-6.85735\times10^{-8}\,i \\ 0.-4.61932\times10^{-8}\,i & 4.04108\times10^{-9} & -0.000059158+6.85735\times10^{-8}\,i & 3.49968\times10^{-9} \end{pmatrix}$$

$$\begin{pmatrix} 6.0972\times10^{-7} & -5.8437\times10^{-10}+4.97101\times10^{-7}\,i & 1.79217\times10^{-7}+3.82069\times10^{-10}\,i & 0.000780844+1.43432\times10^{-6}\,i \\ -5.8437\times10^{-10}-4.97101\times10^{-7}\,i & 4.05284\times10^{-7} & 1.39733\times10^{-10}-1.46115\times10^{-7}\,i & 4.2101\times10^{-7}-0.000636619\,i \\ 1.79217\times10^{-7}-3.82069\times10^{-10}\,i & 1.39733\times10^{-10}+1.46115\times10^{-7}\,i & 5.26781\times10^{-8} & 0.000229517-6.77073\times10^{-8}\,i \\ 0.000780844-1.43432\times10^{-6}\,i & 4.2101\times10^{-7}+0.000636619\,i & 0.000229517+6.77073\times10^{-8}\,i & 0.999999 \end{pmatrix}$$

It is suitable to check the changes in the final state for situations with $\alpha_j = \pm\pi/2$, when phases of the second and third pulses are perpendicular to that of the first pulse. The density operators below are ρ_3 for $\alpha_2 = 0$, and $\alpha_3 = +\pi/2$ (upper variant) and $\alpha_3 = -\pi/2$ (lower variant):

$$\begin{pmatrix} 6.09743\times10^{-7} & -4.4383\times10^{-7}+5.2636\times10^{-8}\,i & 2.25548\times10^{-7}-4.57181\times10^{-8}\,i & 1.29249\times10^{-6}-0.000780859\,i \\ -4.4383\times10^{-7}-5.2636\times10^{-8}\,i & 3.27606\times10^{-7} & -1.68122\times10^{-7}+1.38077\times10^{-8}\,i & -0.0000683483+0.000568273\,i \\ 2.25548\times10^{-7}+4.57181\times10^{-8}\,i & -1.68122\times10^{-7}-1.38077\times10^{-8}\,i & 8.68597\times10^{-8} & 0.0000590264-0.000288748\,i \\ 1.29249\times10^{-6}+0.000780859\,i & -0.0000683483-0.000568273\,i & 0.0000590264+0.000288748\,i & 0.999999 \end{pmatrix}$$

$$\begin{pmatrix} 6.09697\times10^{-7} & 4.43693\times10^{-7}+5.40429\times10^{-8}\,i & 2.25272\times10^{-7}+4.66681\times10^{-8}\,i & -1.29239\times10^{-6}+0.000780829\,i \\ 4.43693\times10^{-7}-5.40429\times10^{-8}\,i & 3.27678\times10^{-7} & 1.68074\times10^{-7}+1.39938\times10^{-8}\,i & 0.0000682714+0.000568346\,i \\ 2.25272\times10^{-7}-4.66681\times10^{-8}\,i & 1.68074\times10^{-7}-1.39938\times10^{-8}\,i & 8.68062\times10^{-8} & 0.0000592896+0.000288602\,i \\ -1.29239\times10^{-6}-0.000780829\,i & 0.0000682714-0.000568346\,i & 0.0000592896-0.000288602\,i & 0.999999 \end{pmatrix}$$

The next pair of system states are ρ_3 for $\alpha_2 = \pi/2$, and $\alpha_3 = \pi/2$ (upper variant) and $\alpha_3 = -\pi/2$ (lower variant):

$$\begin{pmatrix} 3.25367\times10^{-7} & 2.59619\times10^{-7}+1.97833\times10^{-7}\,i & -1.12805\times10^{-7}-6.64752\times10^{-8}\,i & -0.000289428+0.000491527\,i \\ 2.59619\times10^{-7}-1.97833\times10^{-7}\,i & 3.27445\times10^{-7} & -1.30429\times10^{-7}+1.5547\times10^{-8}\,i & 0.0000679219+0.000568183\,i \\ -1.12805\times10^{-7}+6.64752\times10^{-8}\,i & -1.30429\times10^{-7}-1.5547\times10^{-8}\,i & 5.26913\times10^{-8} & -7.78501\times10^{-8}-0.000229546\,i \\ -0.000289428-0.000491527\,i & 0.0000679219-0.000568183\,i & -7.78501\times10^{-8}+0.000229546\,i & 0.999999 \end{pmatrix}$$

$$\begin{pmatrix} 3.25338\times10^{-7} & -2.99164\times10^{-7}-1.30793\times10^{-7}\,i & -1.70891\times10^{-7}-1.00755\times10^{-7}\,i & 0.000289428-0.000491497\,i \\ -2.99164\times10^{-7}+1.30793\times10^{-7}\,i & 3.27677\times10^{-7} & 1.97648\times10^{-7}+2.39473\times10^{-8}\,i & -0.0000685499+0.000568311\,i \\ -1.70891\times10^{-7}+1.00755\times10^{-7}\,i & 1.97648\times10^{-7}-2.39473\times10^{-8}\,i & 1.20968\times10^{-7} & 1.8536\times10^{-7}+0.000347804\,i \\ 0.000289428+0.000491497\,i & -0.0000685499-0.000568311\,i & 1.8536\times10^{-7}-0.000347804\,i & 0.999999 \end{pmatrix}$$

In all of these cases, switching of the SMM states works with high accuracy and the coherences created have amplitudes less than $|x|$. Thus, the relative phases of the pulse fields are of no importance when the amplitudes of those fields are small compared with D. The ZeFIR double resonance technique is a promising variant of SMM switching.

Part C: Geometry Scheme Supposed for Far Infrared SMM Irradiation

Here, we illustrate the scheme of irradiation of the supposed SMM for ZeFIR. Right-handed and left-handed irradiation is shown in Figure S1.

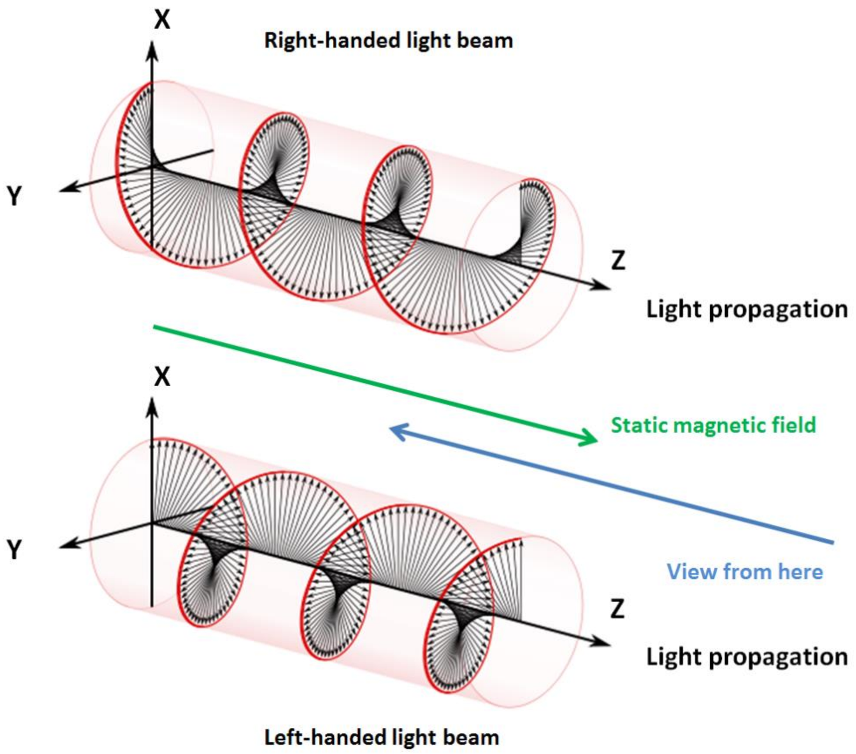

Figure S1. Geometry of SMM irradiation. The right-handed [4] FIR radiation propagates along the Z axis, which is parallel to the symmetry axis of the SMM molecule in the lab frame. The external magnetic field is directed also along the SMM symmetry axis parallel to the radiation propagation direction. An observer looking from the +Z direction, as shown in the picture, would see the magnetic moment or the magnetic field of the radiation rotate counter clockwise if they have right-handed circular polarization, but clockwise if they have left-handed circular polarization. Radiation having the same frequency and the same handedness as a SMM transition can coherently transfer or transmute populations between the energy levels of that SMM transition. The picture was adopted from the work of [5].

References

1. Blum, K. *Density Matrix Theory and Applications.* Springer-Verlag Berlin Heidelberg: Heidelberg, Germany, 2012. DOI 10.1007/978-3-642-20561-3.
2. Schweiger, A.; Jeschke G. Principles of pulse electron paramagnetic resonance. Oxford University Press: Oxford, UK, 2001.
3. Ernst, R.R.; Bodenhausen, G.; Wokaun, A. Principles of Nuclear Magnetic Resonance in One and Two Dimensions. Clarendon Press: Oxford, UK, 1987.
4. Feynman, R.P.; Leighton, R.B.; Sands, M. *The Feynman Lectures on Physics, boxed set: The New Millennium Edition*; Basic Books: New York, NY, USA, 2011; Volume 1, Chapter 33-1, ISBN-13: 978-0465023820.
5. Wikipedia: Circular polarization. Available online: https://en.wikipedia.org/wiki/Circular_polarization (accessed on 19 November 2019).
